# TyG × waist circumference composite indicator and cardiovascular disease risk in older adults across multiple regions: a cross-sectional study

**DOI:** 10.3389/fendo.2025.1687289

**Published:** 2025-10-22

**Authors:** Ying Guo, Jietao Zhang, Tong Chen, Yan Wang, Fanqi Geng, Hongjian Jia

**Affiliations:** ^1^ The Affiliated Hospital of Jining Medical University, Jining, Shandong, China; ^2^ The Affiliated Hospital of Qingdao University, Qingdao, Shandong, China

**Keywords:** triglyceride–glucose index (TyG index), waist circumference, cardiovascular disease, elderly population, insulin resistance, risk prediction

## Abstract

**Objective:**

To investigate the association between the triglyceride-glucose index combined with waist circumference (TyG×WC) and cardiovascular disease (CVD) risk in older adults across multiple populations.

**Methods:**

This study utilized data from three population sources: NHANES (2011–2018), a Chinese community cohort, and a tertiary hospital, enrolling a total of 3,443 eligible older adults. The TyG index was calculated as ln [fasting triglycerides (mg/dL) × fasting glucose (mg/dL)/2], and then multiplied by waist circumference (WC). The resulting TyG×WC values were standardized using z-score normalization and subsequently categorized into quartiles. Cardiovascular disease (CVD) status was used as the outcome variable. Multivariable logistic regression models were constructed to evaluate the association between TyG×WC and CVD risk. Trend tests and subgroup analyses by sex and region were also performed. Model performance was assessed using receiver operating characteristic (ROC) curves, the DeLong test, net reclassification improvement (NRI), integrated discrimination improvement (IDI), Brier score, and 10-fold cross-validation. Clinical utility was evaluated through decision curve analysis (DCA), while E-value analysis was used to estimate the potential impact of unmeasured confounding. The trend effect across the three populations was synthesized using random-effects meta-analysis to assess heterogeneity.

**Results:**

A total of 3,443 participants were included: 1,684 from NHANES (48.91%), 1,263 hospitalized patients from a tertiary hospital (36.68%), and 496 from a community cohort (14.41%). Significant differences were observed across regions in age, TG, TC, LDL, HDL, FPG, ACR, HbA1c, BMI, WC, uric acid, TyG, gender, and CVD prevalence. Multivariable logistic regression indicated a significant positive association between the TyG×WC index and CVD risk. After adjusting for confounders, participants in Q3 and Q4 had significantly higher CVD risk (OR = 1.94 and 2.47, respectively; both P<0.001), with a significant linear trend (P for trend = 2.44×10^-19^). Subgroup analyses showed a stronger predictive effect in females (Q4 vs Q1: OR = 2.34, 95% CI: 1.75–3.14) and in the NHANES population (Q4 vs Q1: OR = 4.64, 95% CI: 3.19–6.85). Heterogeneity analysis revealed no significant differences across regions (I²=30.3%, P = 0.238). Regarding model performance, the extended model including TyG×WC showed an improvement in AUC (from 0.692 to 0.701, DeLong P = 0.038), along with significant improvements in NRI (0.222, P<0.001), IDI (0.0215, P<0.001), and favorable DCA results. The E-value analysis indicated robust results against unmeasured confounding (point estimate E-value = 4.27; lower bound E-value = 3.29).

**Conclusion:**

The TyG×WC composite indicator is an independent predictor of CVD risk, with more pronounced effects observed in women and the general population. The association between TyG×WC and CVD risk demonstrates a stable and progressive trend across quartiles and is consistent across different populations. The inclusion of TyG×WC enhances predictive accuracy (AUC, NRI, IDI) and clinical utility (DCA), suggesting strong generalizability and practical application. This indicator may serve as a valuable tool for screening high-risk individuals and guiding CVD prevention strategies.

## 1 Introduction

Cardiovascular disease (CVD) remains the leading cause of death and disability worldwide. With the rapid aging of the population and urbanization, the number of individuals affected by CVD continues to rise (1). Early identification of high-risk individuals is critical for timely intervention and effective prevention strategies. In 2015, the total direct medical cost of CVD was approximately $318 billion, and this figure is projected to rise to $749 billion by 2035. It is also estimated that by 2035, 45.1% of the U.S. population will be affected by some form of cardiovascular disease, with an even higher proportion expected among the elderly population (2). Abnormal glucose and lipid metabolism is highly prevalent in individuals with cardiovascular disease and has been strongly linked to poor clinical prognosis (3). Therefore, effective management of glucose and lipid metabolism is essential both for the prevention of CVD and for improving outcomes in patients with established CVD.

Insulin resistance (IR) refers to a physiological state in which the body’s sensitivity and responsiveness to insulin are reduced. IR is recognized as one of the contributing factors to cardiovascular disease (CVD) (4, 5) and is closely intertwined with abnormalities in glucose and lipid metabolism. The triglyceride-glucose (TyG) index, calculated from fasting plasma glucose (FPG) and triglyceride (TG) levels, is a composite metabolic marker that has been widely used as a surrogate for assessing insulin resistance (4). Compared with direct measures such as fasting insulin, the TyG index is simpler, more economical, and better suited for large-scale clinical application. Previous studies have demonstrated that the TyG index is significantly associated with the prediction, progression, prognosis, and mortality of CVD (6–8). Obesity is also an important risk factor for CVD, strongly associated with both disease onset and clinical outcomes. In epidemiological research, overweight and obesity are often assessed using body mass index (BMI). However, BMI has limited sensitivity and is susceptible to various confounding factors (9),such as ethnicity, sex, age, and socioeconomic status. Waist circumference (WC), in contrast, has been shown to better reflect body fat distribution (10), and has superior predictive value for CVD (11). While both TyG and WC individually predict CVD risk, each has limitations in sensitivity and specificity when used alone.

Previous studies have attempted to combine metabolism with other indicators (12–14), such as TyG-BMI and TyG-AIP, to improve predictive performance. However, research on the use of the TyG index multiplied by waist circumference (TyG×WC) as a composite predictor for CVD remains limited, particularly in general and high-risk populations.

Using data from the U.S. National Health and Nutrition Examination Survey (NHANES), Qiu et al. demonstrated that the triglyceride–glucose index multiplied by waist circumference (TyG×WC) provides superior predictive ability for cardiovascular disease (CVD) mortality and diagnostic discrimination compared with the TyG index alone. In another independent NHANES analysis, Dang et al. similarly confirmed the incremental value of this composite indicator over conventional TyG-related parameters (15). Evidence from Chinese cohorts further supports this finding: Zhu et al. reported that longitudinal changes in TyG×WC were more effective in identifying individuals at high risk of CVD (16), while Yu et al. showed that both TyG×WC and TyG-BMI were strong predictors of CVD outcomes. Consistently (17), Zhu et al. suggested that TyG×WC may have greater clinical relevance than other TyG-based measures. Collectively, these studies highlight TyG-derived composite indices particularly TyG×WC as practical and robust tools for CVD risk stratification and mortality prediction.

Evidence is especially scarce regarding its application in Chinese community and hospitalized cohorts. Furthermore, there is a lack of comprehensive evaluation of this index in terms of stability, generalizability, and clinical utility, including decision curve analysis (DCA) and reclassification metrics. To address these gaps, the present study integrates data from three population sources—NHANES, a Chinese community cohort, and a hospital cohort to construct and validate the predictive value of the TyG×WC index for CVD risk. By applying a multidimensional modeling approach including ROC analysis, NRI/IDI, cross-validation, E-value analysis, and heterogeneity meta-analysis, we systematically evaluate the independence, robustness, and clinical applicability of this composite indicator. The goal is to provide a low-cost, accessible tool for early identification and stratified management of CVD risk.

## 2 Methods

### 2.1 Study population

**Figure 1:**
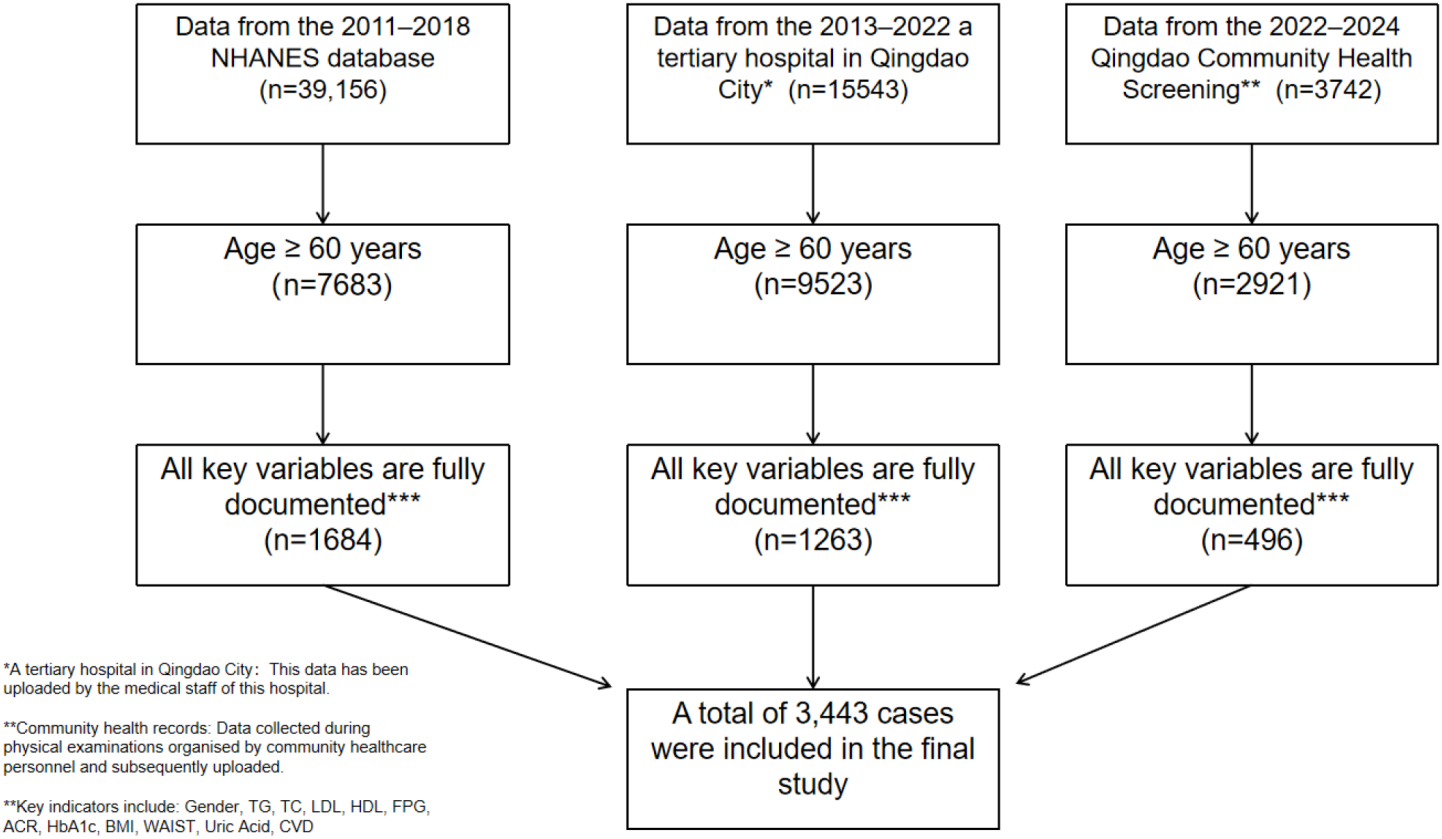
Study flow diagram for participant selection across datasets.

This multi-dataset study enrolled 3,443 older adults from three independent sources: the US National Health and Nutrition Examination Survey (2011–2018), an inpatient registry from a tertiary hospital in China (2013–2022), and a Chinese community health-screening cohort (2022–2024). Eligibility required age ≥60 years and complete records for key variables. Participants were categorized by data source: NHANES (region 1; n=1,684), hospital (region 2; n=1,263), and community (region 3; n=496). For specific details, see **Figure 1**.

### 2.2 Data collection and variable definitions

Demographic characteristics (e.g., age, sex), clinical laboratory indicators (including triglycerides [TG], total cholesterol [TC], low-density lipoprotein cholesterol [LDL], high-density lipoprotein cholesterol [HDL], fasting plasma glucose [FPG], glycated hemoglobin [HbA1c], uric acid [UA], and urinary albumin-to-creatinine ratio [ACR]), and anthropometric measures (body mass index [BMI] and waist circumference [WC]) were collected for all participants.

The TyG index was calculated using the following internationally accepted formula:

TG:1 mg/dL≈0.01129 mmol/L; FPG:1 mg/dL≈0.0555 mmol/L
TyG index = ln [TG (mg/dL) × FPG (mg/dL)/2]

To ensure consistency in WC classification across populations from different countries/regions, region-specific criteria were used to define abdominal obesity. For the NHANES population, central obesity was defined as WC ≥ 102 cm for men and ≥ 88 cm for women (10). For the Chinese community and hospital populations, thresholds of WC ≥ 90 cm for men and ≥ 80 cm for women were applied (18).

Cardiovascular disease (CVD) was defined based on documented clinical history, including coronary heart disease, stroke, or heart failure, as recorded in each dataset.

### 2.3 Calculation and categorization of the TyG×WC composite index

To eliminate differences in scale across datasets, both the TyG index and WC were standardized using Z-score transformation:

Z = (raw value − mean)/standard deviation.

The product of the standardized TyG and WC values (TyG_z × WC_z) was computed to represent a combined metabolic–adiposity exposure risk. This composite index was then categorized into quartiles, designated as Q1, Q2, Q3, and Q4.

### 2.4 Statistical analysis

Normality of continuous variables was assessed using the Shapiro–Wilk test. For variables that deviated from normality, two-group comparisons were performed with the Mann–Whitney U test, and ≥3-group comparisons with the Kruskal–Wallis test (with Dunn–Bonferroni *post-hoc* tests when applicable). Categorical variables were compared using the χ² test. Continuous variables were expressed as mean ± standard deviation (SD), and categorical variables as counts and percentages. Group comparisons were conducted using ANOVA or the chi-square test as appropriate. Multivariable logistic regression models were used to assess the association between TyG×WC quartiles and CVD, adjusting for all covariates. For trend analysis, the TyG×WC quartile variable was treated as an ordinal variable. Subgroup analyses were performed by sex and by region, and the trend regression coefficients across regions were pooled using a random-effects meta-analysis. Heterogeneity was assessed using Cochran’s Q test and the I² statistic.

To limit data-driven bias, we used prior knowledge plus purposeful selection. We prespecified *a priori* covariates for the Standard model—age, sex, region (NHANES/hospital/community), BMI, HDL-C, total cholesterol, HbA1c, and uric acid, to account for demographic, contextual, and metabolic confounding. We then applied a purposeful selection procedure: variables with univariable P<0.10 were considered as candidates, but retention was primarily based on a ≥10% change-in-estimate criterion for the key effect measure and clinical plausibility rather than statistical significance alone. Multicollinearity was assessed with VIF<5.

Model performance was evaluated by comparing a baseline model (with covariates only) and an extended model (including TyG×WC), using the area under the receiver operating characteristic curve (AUC) and the DeLong test. Net reclassification improvement (NRI), integrated discrimination improvement (IDI), Brier score, and 10-fold cross-validation were used to further evaluate predictive performance. Decision curve analysis (DCA) was applied to assess clinical utility across a range of risk thresholds. To assess the robustness of the TyG×WC effect against unmeasured confounding, E-value analysis was conducted.

Analyses were performed in R (version 4.3.3, Windows x64). Key packages (version) were: rms (8.0.0) for regression modeling and calibration, pROC (1.18.5) for ROC/AUC with DeLong CIs, ggplot2 (3.5.2) for figure generation, car (3.1.3) for variance inflation factors (VIF), ResourceSelection (0.3.6) for the Hosmer–Lemeshow test, rmda (1.6) for decision curve analysis, nricens (1.6) for reclassification (NRI/IDI), and dplyr (1.1.4), tidyr (1.3.1), readxl (1.4.5), writexl (1.5.4), broom (1.0.8) for data handling and report-ready outputs. A two-sided P value < 0.05 was considered statistically significant.

### 2.5 Ethical considerations

The NHANES dataset is publicly available and fully de-identified; therefore, no additional ethical approval was required. For the hospital and community datasets from China, the study protocol was approved by the institutional ethics committee, and the requirement for informed consent was waived due to the retrospective design. All personal identifiers were removed prior to data analysis.

## 3 Results

### 3.1 Baseline characteristics

A total of 3,443 participants were included in this study, originating from three sources: NHANES data (Region 1), hospitalized patients (Region 2), and community-based screening (Region 3). Participants were evenly distributed across the TyG×WC quartiles: 861 individuals (25.01%) in quartile 1 (Q1), 861 (25.01%) in Q2, 860 (24.98%) in Q3, and 861 (25.01%) in Q4.As shown in **Table 1**, significant differences were observed among the four quartile groups with respect to age, triglycerides (TG, mmol/L), total cholesterol (TC), low-density lipoprotein (LDL), high-density lipoprotein (HDL), fasting plasma glucose (FPG, mmol/L), albumin-to-creatinine ratio (ACR), glycated hemoglobin (HbA1c), body mass index (BMI), waist circumference (WC), uric acid, TyG index, and the prevalence of cardiovascular disease (CVD) (P < 0.05 for all). In contrast, there was no statistically significant difference in gender distribution across the groups (P > 0.05).

**Table 1:** Baseline characteristics of participants according to TyG×WC quartiles.

Variables	Total (n = 3443)	Q1 (n = 861)	Q2 (n = 861)	Q3 (n = 860)	Q4 (n = 861)	Statistic	*P*
Age, Mean ± SD	71.20 ± 6.52	71.86 ± 6.03	71.33 ± 6.55	71.10 ± 6.55	70.50 ± 6.87	F=6.47	**<.001**
TG, Mean ± SD	1.63 ± 1.12	1.99 ± 1.32	1.48 ± 0.70	1.44 ± 0.66	1.62 ± 1.47	F=44.45	**<.001**
TC, Mean ± SD	4.76 ± 1.24	4.99 ± 1.36	4.66 ± 1.19	4.69 ± 1.15	4.69 ± 1.20	F=13.78	**<.001**
LDL, Mean ± SD	2.64 ± 0.93	2.68 ± 0.94	2.65 ± 0.93	2.69 ± 0.93	2.53 ± 0.92	F=5.12	**0.002**
HDL, Mean ± SD	1.48 ± 0.65	1.44 ± 0.52	1.46 ± 0.65	1.52 ± 0.73	1.50 ± 0.69	F=2.85	**0.036**
FPG, Mean ± SD	7.00 ± 2.57	8.14 ± 3.17	6.59 ± 1.83	6.34 ± 1.66	6.91 ± 2.91	F=89.46	**<.001**
ACR, Mean ± SD	57.63 ± 250.33	77.21 ± 319.24	41.95 ± 124.18	56.54 ± 263.46	54.82 ± 251.99	F=2.93	**0.032**
HbA1c, Mean ± SD	6.58 ± 1.36	6.98 ± 1.60	6.43 ± 1.15	6.37 ± 1.12	6.55 ± 1.43	F=36.84	**<.001**
BMI, Mean ± SD	27.23 ± 5.10	27.07 ± 5.52	27.53 ± 3.59	26.79 ± 3.62	27.55 ± 6.87	F=4.56	**0.003**
WAIST, Mean ± SD	94.40 ± 14.96	91.72 ± 18.11	95.83 ± 10.11	94.32 ± 9.31	95.75 ± 19.17	F=14.38	**<.001**
Uric Acid, Mean ± SD	329.77 ± 90.22	336.52 ± 90.45	334.46 ± 84.01	323.49 ± 87.32	324.58 ± 97.93	F=4.73	**0.003**
TyG, Mean ± SD	8.90 ± 0.75	9.20 ± 0.90	8.83 ± 0.52	8.78 ± 0.48	8.78 ± 0.90	F=65.78	**<.001**
Gender, n(%)						χ²=4.38	0.223
Female	1891 (54.92)	469 (54.47)	454 (52.73)	496 (57.67)	472 (54.82)		
Male	1552 (45.08)	392 (45.53)	407 (47.27)	364 (42.33)	389 (45.18)		
CVD, n(%)						χ²=50.55	**<.001**
NO	2062 (59.89)	547 (63.53)	575 (66.78)	500 (58.14)	440 (51.10)		
YES	1381 (40.11)	314 (36.47)	286 (33.22)	360 (41.86)	421 (48.90)		

TG, triglycerides (mmol/L); TC, total cholesterol (mmol/L); LDL-C, low-density lipoprotein cholesterol (mmol/L); HDL-C, high-density lipoprotein cholesterol (mmol/L); FPG, fasting plasma glucose (mmol/L); ACR, urinary albumin-to-creatinine ratio (mg/g); HbA1c, glycated hemoglobin A1c (%); BMI, body mass index (kg/m²); WC, waist circumference (cm); UA, uric acid (µmol/L); TyG, triglyceride–glucose index (unitless); CVD, cardiovascular disease (yes/no); SD, standard deviation.

F, analysis of variance; χ², chi-square test. F: ANOVA, χ², Chi-square test; SD, standard deviation.

The bolded text in the article indicates statistical significance.

Among the participants, 1,684 individuals (48.91%) were from Region 1 (NHANES), 1,263 (36.68%) from Region 2 (hospitalized patients), and 496 (14.41%) from Region 3 (community-based screening). Significant differences among the three regions were observed in the following variables: age, triglycerides (TG, mmol/L), total cholesterol (TC), low-density lipoprotein (LDL), high-density lipoprotein (HDL), fasting plasma glucose (FPG, mmol/L), albumin-to-creatinine ratio (ACR), glycated hemoglobin (HbA1c), body mass index (BMI), waist circumference (WC), uric acid, TyG index, gender, CVD prevalence, and TyG×WC quartile distribution (P < 0.05 for all). Comparisons among regions are listed in **Table 2**.

**Table 2:** Baseline characteristics of participants by population source (NHANES, hospital, community).

Variables	Region 1 (n = 1684)	Region 2 (n = 1263)	Region 3 (n = 496)	Statistic	*P*
Age, Mean ± SD	70.40 ± 6.89	72.70 ± 5.23	70.08 ± 7.44	F=54.75	**<.001**
TG, Mean ± SD	1.38 ± 0.98	2.14 ± 1.20	1.21 ± 0.82	F=240.06	**<.001**
TC, Mean ± SD	4.88 ± 1.10	4.82 ± 1.41	4.17 ± 1.03	F=67.49	**<.001**
LDL, Mean ± SD	2.79 ± 0.93	2.54 ± 0.93	2.38 ± 0.83	F=50.49	**<.001**
HDL, Mean ± SD	1.46 ± 0.47	1.15 ± 0.42	2.38 ± 0.83	F=993.20	**<.001**
FPG, Mean ± SD	6.65 ± 2.29	8.19 ± 2.83	5.13 ± 0.57	F=335.49	**<.001**
ACR, Mean ± SD	43.89 ± 107.38	72.80 ± 283.71	65.66 ± 435.91	F=5.12	**0.006**
HbA1c, Mean ± SD	6.17 ± 1.26	7.36 ± 1.39	5.99 ± 0.32	F=407.05	**<.001**
BMI, Mean ± SD	28.78 ± 6.05	26.05 ± 3.29	24.98 ± 3.42	F=175.09	**<.001**
WAIST, Mean ± SD	101.74 ± 14.72	85.47 ± 10.86	92.24 ± 11.27	F=578.44	**<.001**
Uric Acid, Mean ± SD	335.20 ± 86.97	324.19 ± 92.87	325.51 ± 93.25	F=6.03	**0.002**
TyG, Mean ± SD	8.71 ± 0.64	9.36 ± 0.69	8.36 ± 0.54	F=570.11	**<.001**
Gender, n(%)				χ²=10.08	**0.006**
Female	899 (53.38)	737 (58.35)	255 (51.41)		
Male	785 (46.62)	526 (41.65)	241 (48.59)		
CVD, n(%)				χ²=68.71	**<.001**
NO	1056 (62.71)	652 (51.62)	354 (71.37)		
YES	628 (37.29)	611 (48.38)	142 (28.63)		
TyG WAIST, n(%)				χ²=300.90	**<.001**
Q1	293 (17.40)	512 (40.54)	56 (11.29)		
Q2	415 (24.64)	317 (25.10)	129 (26.01)		
Q3	479 (28.44)	234 (18.53)	147 (29.64)		
Q4	497 (29.51)	200 (15.84)	164 (33.06)		

TG, triglycerides (mmol/L); TC, total cholesterol (mmol/L); LDL-C, low-density lipoprotein cholesterol (mmol/L); HDL-C, high-density lipoprotein cholesterol (mmol/L); FPG, fasting plasma glucose (mmol/L); ACR, urinary albumin-to-creatinine ratio (mg/g); HbA1c, glycated hemoglobin A1c (%); BMI, body mass index (kg/m²); WC, waist circumference (cm); UA, uric acid (µmol/L); TyG, triglyceride–glucose index (unitless); CVD, cardiovascular disease (yes/no).

F, analysis of variance; χ², chi-square test.

The bolded text in the article indicates statistical significance.

### 3.2 Association between TyG×WC and CVD risk

**Figure 2:**
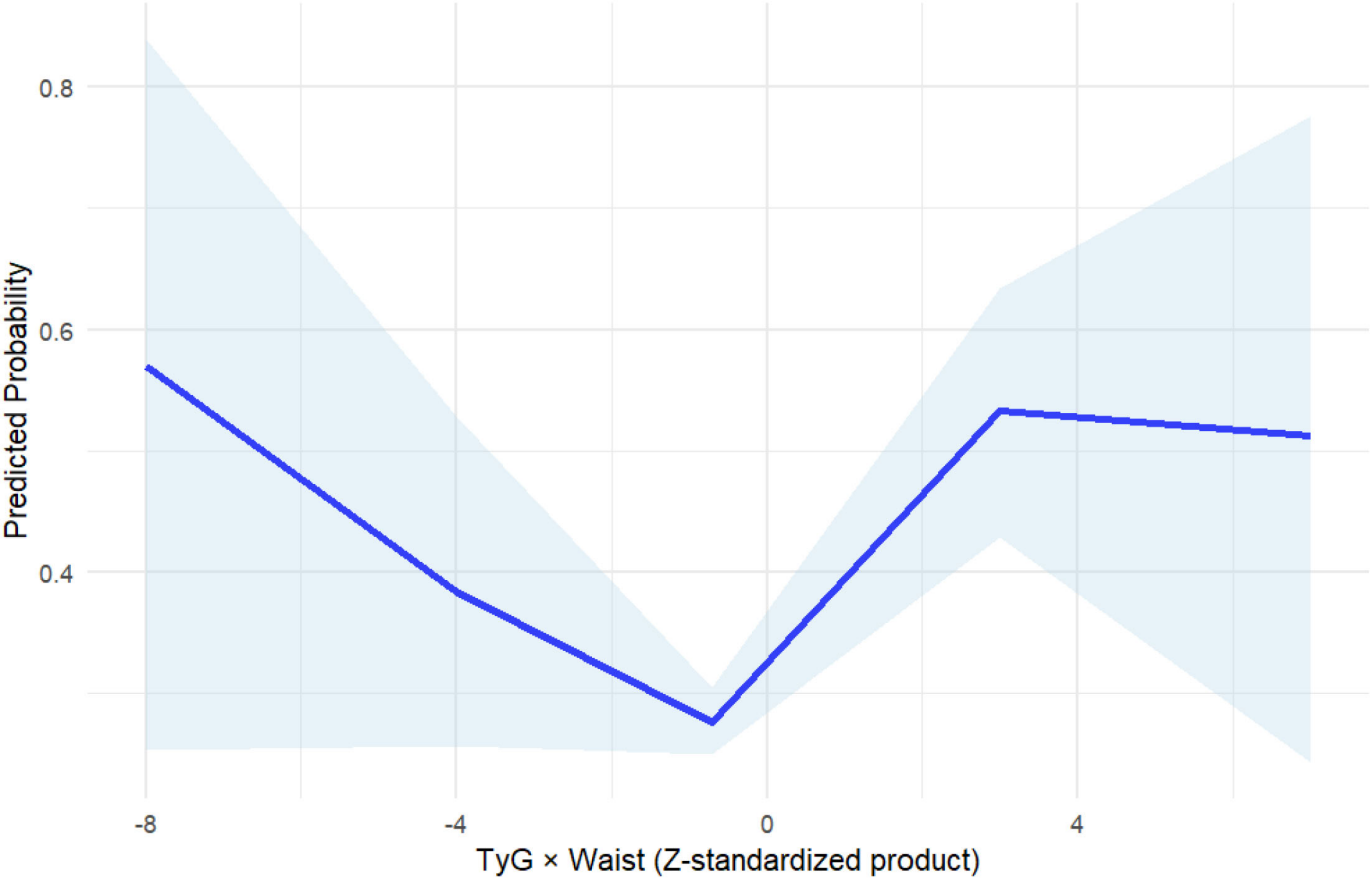
Restricted cubic spline (RCS) analysis of TyG×WC and cardiovascular disease risk. The blue solid line represents the odds ratio (OR) for cardiovascular disease (CVD) across continuous values of TyG×WC, and the shaded area indicates the 95% confidence interval (CI).

As can be seen in **Table 3**, in the logistic regression models incorporating covariates such as sex, age, lipid profile, glycemic indicators, and BMI, the first quartile (Q1) of the TyG×WC index was used as the reference group to assess its association with cardiovascular disease (CVD). Univariate analysis revealed a strong linear relationship between higher TyG×WC levels and increased CVD risk. The unadjusted odds ratios (ORs) for Q3 and Q4 were 1.25 (95% CI: 1.03–1.52) and 1.67 (95% CI: 1.37–2.02), respectively (P < 0.05 for both).In the multivariable-adjusted model, after controlling for potential confounders including sex, total cholesterol (TC), high-density lipoprotein cholesterol (HDL-C), HbA1c, and BMI, elevated TyG×WC levels remained independently associated with CVD. The adjusted OR for Q3 was 1.94 (95% CI: 1.56–2.41), and for Q4 was 2.47 (95% CI: 1.98–3.09), with both P values < 0.001. A significant linear trend was observed across quartiles (Ptrend = 2.44×10^-19^). As shown in **Figure 2**, restricted cubic spline (RCS) regression further confirmed a monotonic non-U-shaped dose-response relationship.

**Table 3:** Association between TyG×WC quartiles and cardiovascular disease risk in logistic regression models.

Variables	Univariable P	Univariable OR (95% CI)	Multivariable P	Multivariable OR (95% CI)
TyG×WC Q2 vs. Q1	0.157	0.87 (0.71–1.06)	0.422	1.09 (0.88–1.35)
TyG×WC Q3 vs. Q1	0.022	1.25 (1.03–1.52)	<0.001	1.94 (1.56–2.41)
TyG×WC Q4 vs. Q1	<0.001	1.67 (1.37–2.02)	<0.001	2.47 (1.98–3.09)
Gender (Female vs. Male)	<0.001	0.79 (0.69–0.90)	0.019	0.82 (0.70–0.97)
TC	<0.001	1.18 (1.11–1.24)	<0.001	1.30 (1.21–1.39)
HDL	<0.001	0.62 (0.55–0.69)	<0.001	0.68 (0.57–0.81)
HbA1c	<0.001	1.29 (1.22–1.36)	<0.001	1.18 (1.11–1.26)
BMI	<0.001	1.10 (1.08–1.11)	<0.001	1.11 (1.09–1.13)
Uric Acid	<0.001	1.01 (1.01–1.01)	0.002	1.01 (1.01–1.01)
Region 2 vs. 1	<0.001	1.58 (1.36–1.83)	<0.001	2.00 (1.64–2.43)
Region 3 vs. 1	<0.001	0.67 (0.54–0.84)	<0.001	1.78 (1.32–2.40)

### 3.3 Stratified and interaction analyses

**Figure 3:**
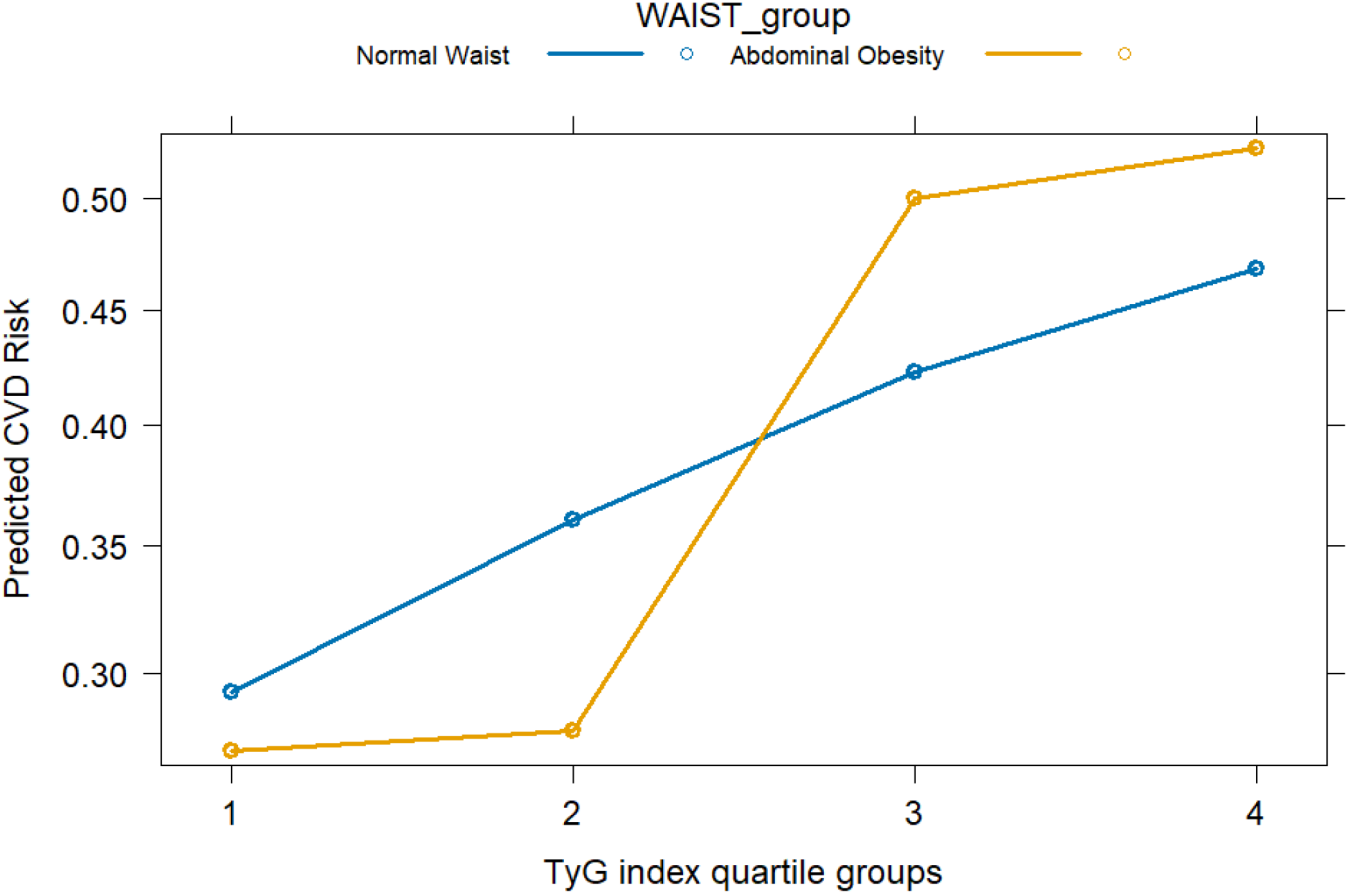
Interaction between TyG index quartiles and waist circumference categories in relation to cardiovascular disease (CVD) risk. CVD risk was evaluated by odds ratios derived from logistic regression models. Waist circumference (WC) was categorized according to region-specific cut-offs for abdominal obesity (NHANES: ≥NHA cm in men, ≥en cm in women; Chinese cohorts: ≥oh cm in men, ≥en cm in women).

To further explore the interaction between the TyG index and waist circumference (WC) on the risk of cardiovascular disease (CVD), an interaction term between TyG quartiles and WC categories was included in the logistic regression model. The results demonstrated a statistically significant interaction (P for interaction < 0.05).As shown in the figure, among individuals with high WC, the risk of CVD increased more markedly with rising TyG levels, suggesting a potential synergistic effect between metabolic status and central adiposity on CVD risk. **Figure 3** illustrates the synergistic interaction between TyG and waist circumference.

**Figure 4:**
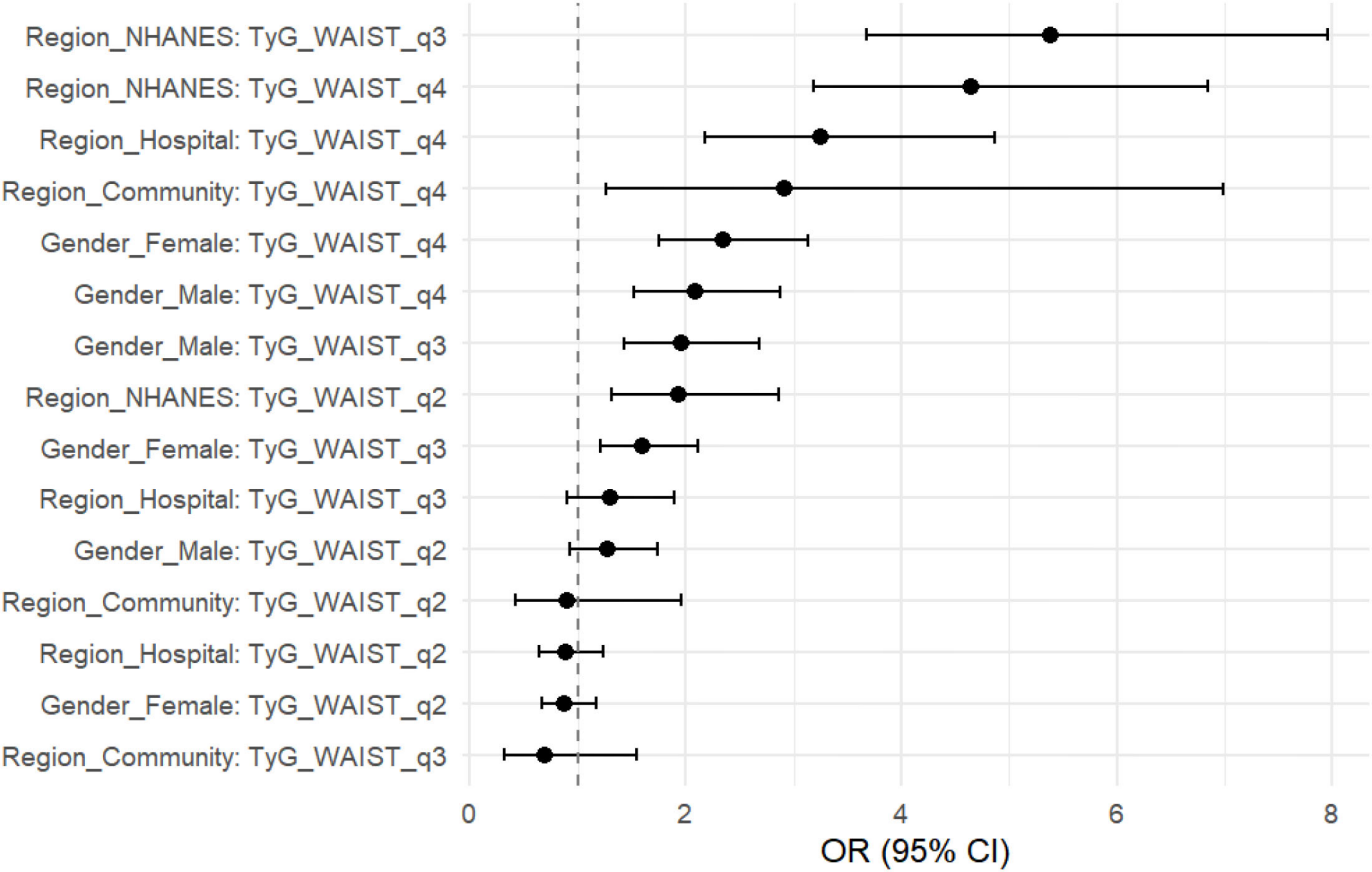
Stratified forest plot of TyG×WC quartiles and CVD risk. Odds ratios (OR) with 95% confidence intervals (CI) are shown for the highest quartile (Q4) compared with the lowest quartile (Q1), stratified by sex (male, female) and population source (NHANES, hospital, community).

To further evaluate the stability and applicability of the TyG×WC composite indicator across different populations, stratified analyses were conducted by sex and region. In **Table 4** and **Figure 4**, the results showed that the predictive effect of this index was more pronounced in females, with an OR of 2.34 (95% CI: 1.75–3.14, P < 0.001) for Q4 compared to Q1, while the corresponding OR in males was 2.09 (95% CI: 1.52–2.87, P < 0.001).Across different regions, the TyG×WC index also showed varying strengths of association with CVD risk. In the NHANES population, the ORs for Q3 and Q4 were 5.38 and 4.64, respectively (both P < 0.001). In the hospital cohort, the ORs were 1.30 and 3.25, while in the community cohort, they were 0.69 and 2.91, respectively. These findings suggest that the predictive strength of the TyG×WC index may differ across subpopulations.

**Table 4:** Stratified logistic regression results for TyG×WC quartiles and CVD risk by sex and region.

Group	Variable	OR (95% CI)	P-value
Gender_Female	Q2	0.88 (0.66–1.17)	0.380
Gender_Female	Q3	1.60 (1.21–2.11)	0.001
Gender_Female	Q4	2.34 (1.75–3.14)	<0.001
Gender_Male	Q2	1.27 (0.93–1.75)	0.132
Gender_Male	Q3	1.95 (1.43–2.69)	<0.001
Gender_Male	Q4	2.09 (1.52–2.87)	<0.001
Region_NHANES	Q2	1.93 (1.32–2.86)	<0.001
Region_NHANES	Q3	5.38 (3.68–7.95)	<0.001
Region_NHANES	Q4	4.64 (3.19–6.85)	<0.001
Region_Hospital	Q2	0.89 (0.64–1.24)	0.483
Region_Hospital	Q3	1.30 (0.90–1.89)	0.165
Region_Hospital	Q4	3.25 (2.18–4.86)	<0.001
Region_Community	Q2	0.90 (0.42–1.96)	0.783
Region_Community	Q3	0.69 (0.32–1.55)	0.367
Region_Community	Q4	2.91 (1.27–6.99)	0.014

To assess whether the differences in effect estimates across populations were statistically significant, we further performed a heterogeneity analysis. A random-effects meta-analysis based on the three populations (NHANES, hospital, and community cohorts) showed no significant heterogeneity in the association between TyG×WC and CVD across regions (Q = 2.87, P = 0.238; I² = 30.3%). The pooled effect estimate demonstrated a significant association, with a combined OR of 1.50 (95% CI: 1.35–1.66), indicating a consistent positive relationship between the TyG×WC composite index and CVD risk across diverse populations.

### 3.4 Predictive performance and incremental value

**Figure 5:**
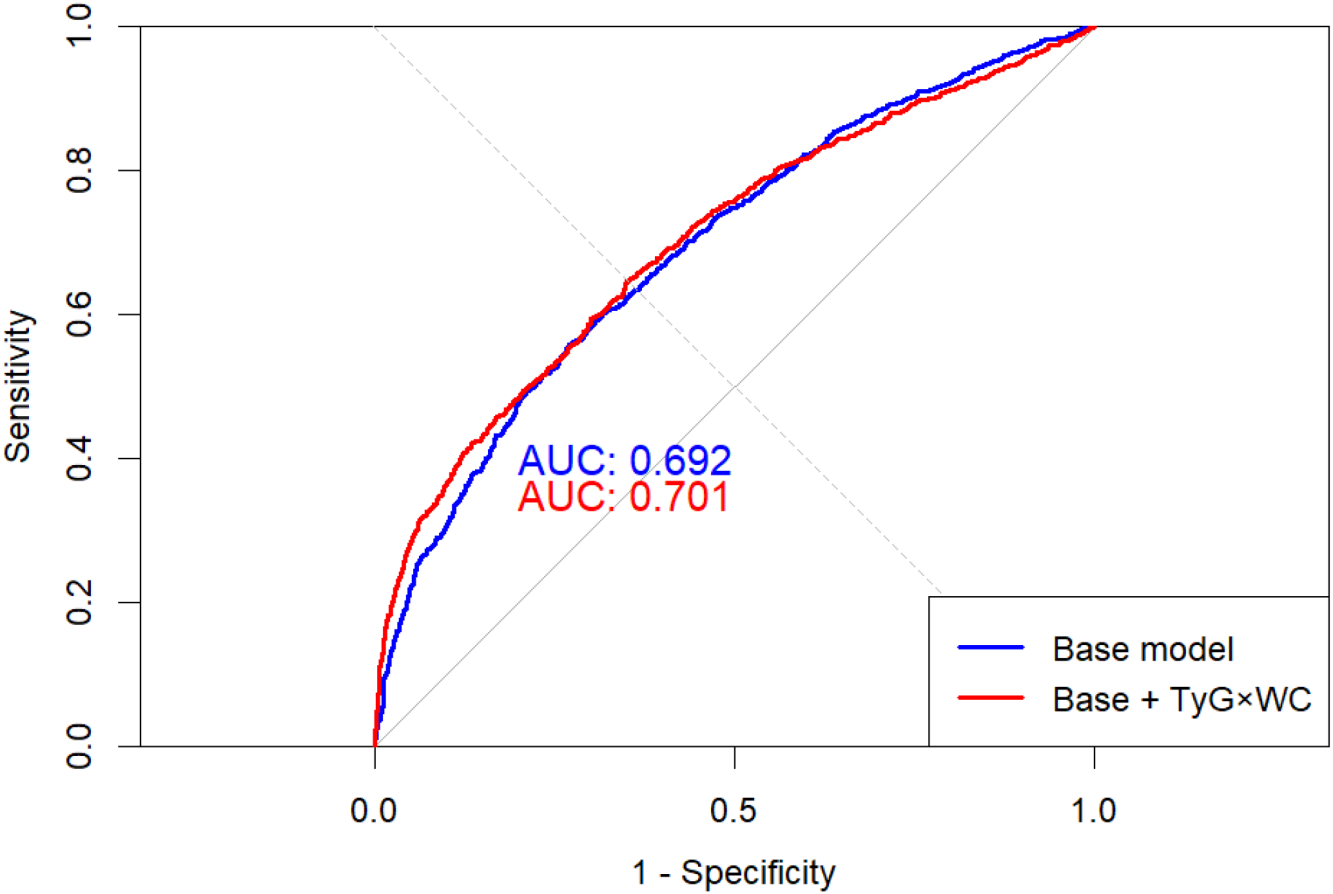
ROC curves of base and extended models for CVD prediction. The red curve represents the baseline model including conventional covariates, and the blue curve represents the extended model additionally incorporating TyG×WC. The dashed diagonal line represents the reference line (AUC = 0.5). The extended model showed a statistically significant improvement in area under the curve (AUC) compared with the baseline model (DeLong test P = 0.038).

Adding TyG×WC to a traditional CVD model significantly improved its performance: in **Figure 5**, AUC increased from 0.692 to 0.701 (DeLong p = 0.038), and as shown in **Table 5** the net reclassification and discrimination indices were NRI = 0.222 (95% CI: 0.155–0.290, p < 0.001) and IDI = 0.0215 (95% CI: 0.0167–0.0262, p < 0.001), respectively. Bootstrap validation yielded an optimism-corrected AUC of 0.706 and a Brier score of 0.2064.

**Table 5:** Predictive performance metrics of the base and extended models.

Metric	Estimate	95% CI	P-value
Overall NRI	0.222	0.155–0.290	<0.001
NRI (Events)	0.133	0.080–0.185	<0.001
NRI (Non-events)	0.090	0.047–0.133	<0.001
IDI	0.0215	0.0167–0.0262	<0.001

OR, odds ratio; CI, confidence interval; NRI, net reclassification improvement; IDI, integrated discrimination improvement; AUC, area under the receiver operating characteristic curve; DCA, decision curve analysis.

### 3.5 Clinical utility and robustness

**Figure 6:**
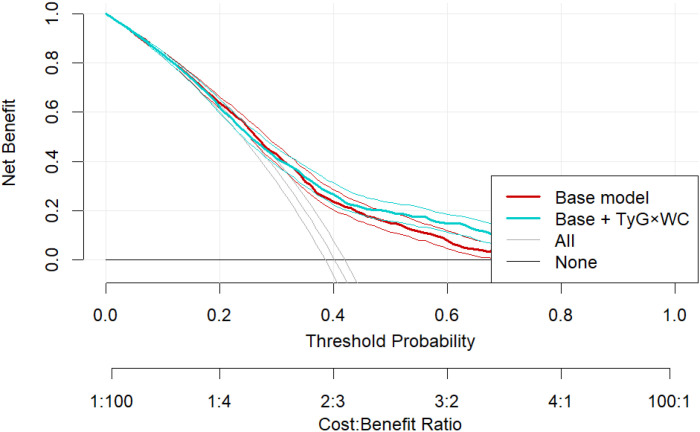
Decision curve analysis comparing the clinical net benefit of the base model and the TyG×WC-extended model across a range of threshold probabilities.

Decision curve analysis (DCA) showed greater net clinical benefit for the TyG×WC-enhanced model within the threshold probability range of 10–35% (**Figure 6**). E-value analysis indicated the OR of Q4 (2.42) corresponded to an E-value of 4.27 (lower CI: 3.29), suggesting robustness to unmeasured confounding. Random-effects meta-analysis across the three subpopulations demonstrated consistent effects (pooled OR = 1.50, 95% CI: 1.35–1.66; I² = 30.3%, p = 0.238), indicating acceptable inter-regional consistency.

## 4 Discussion

Cardiovascular disease (CVD) is the world’s leading cause of death and disability, so the early identification of its risk factors and accurate risk stratification are of great clinical importance. In this multicenter, cross-sectional study of elderly adults, we systematically evaluated the triglyceride–glucose index multiplied by waist circumference (TyG × WC)—a combined metabolic-adiposity marker—across several geographic populations and demonstrated, for the first time, its value in predicting CVD risk. Our findings complement existing evidence on the clinical utility of this joint indicator.

Previous studies have explored the TyG index alone, or in combination with other markers, in relation to CVD events and have reported encouraging results. For instance, Barzegar et al. showed that a higher TyG index was associated with an increased risk of CVD events (19). and several investigations have linked TyG trajectories to CVD progression (20). Moreover, TyG is implicated in diabetes, atherosclerosis, hypertension, liver disease and renal dysfunction (21–23).

In the present analysis, rising TyG × WC levels were associated with a linear increase in CVD risk (P for trend = 2.44 × 10^-19^); participants in the highest quartile (Q4) had a 2.47-fold higher risk of CVD than those in Q1. This relationship was largely consistent across subgroups, and was particularly pronounced among women (OR = 2.34) and in the NHANES cohort (OR = 4.64). A stratified meta-analysis revealed low heterogeneity (I² = 30.3%), underscoring the generalizability of TyG × WC across diverse settings. The convergence of the dose-response trend and subgroup consistency strengthens the indicator’s value across the entire CVD risk continuum.

Mechanistically, the TyG index reflects insulin resistance (IR) (4), IR is a risk factor for CVD, can lead to the development of CVD, and predict cardiovascular outcomes in CVD patients (24). Patients with IR often experience metabolic disorders, with significantly increased risks of hypertension, hyperglycaemia, and dyslipidaemia. Notably, the majority of these abnormalities are risk factors for adverse CVD outcomes (25). As one of the core mechanisms underlying CVD development, the impact of IR involves multiple physiological systems. First, IR induces hyperinsulinemia, leading to dyslipidaemia and exacerbating atherosclerosis (26, 27). Additionally, IR weakens insulin’s ability to activate the PI3K pathway in endothelial cells, inhibits nitric oxide (NO) synthesis, and impairs vascular dilation function, resulting in abnormal vascular reactivity (28, 29). Second, IR activates inflammatory pathways (30), accelerating plaque formation. Additionally, IR increases the risk of thrombosis by upregulating plasminogen activator inhibitor-1 (PAI-1) and enhancing platelet aggregation (31). Waist circumference is a sensitive indicator of central obesity. The product of the two not only quantifies the severity of metabolic disorders but also combines the cumulative effects of visceral fat on insulin signal interference. Women, constrained by postmenopausal fat redistribution and hormonal fluctuations (32, 33), may be more prone to enhanced insulin resistance, which may explain the stronger correlation between TyG and WC in this study.

We further confirmed the incremental value of TyG × WC in CVD-prediction models. After adding TyG × WC, the area under the ROC curve (AUC) rose from 0.692 to 0.701 (DeLong test, P = 0.038); net reclassification improvement (NRI) and integrated discrimination improvement (IDI) also increased significantly and remained stable in 10-fold cross-validation (AUC = 0.711). Decision-curve analysis (DCA) indicated higher net benefit within clinically relevant risk thresholds (10–35%). In addition, the E-value was 4.27, suggesting that a very strong unmeasured confounder would be required to negate the observed association, thereby supporting the robustness of our findings. Collectively, these results indicate that TyG × WC is not only statistically significant but also clinically actionable and broadly applicable.

## 5 Limitations

First, the cross-sectional design precludes causal inference. Second, although we adjusted for major metabolic covariates, we could not stratify by race/ethnicity or comprehensively account for socioeconomic status and educational attainment, which are recognized determinants of both adiposity and cardiometabolic risk; residual confounding is therefore possible. Third, dataset-specific differences in measurement protocols and self-reported components (e.g., CVD history in NHANES) may introduce information bias, despite unified definitions and QA procedures. Fourth, inflammatory biomarkers and fasting insulin were unavailable in the Chinese datasets, limiting mechanistic inferences. These caveats should be considered when interpreting our findings. Future studies could further assess the temporal dynamics of TyG×WC and CVD events in prospective cohorts and explore mediating pathways involving inflammatory factors and arterial stiffness scores.

## Data Availability

The original contributions presented in the study are included in the article/supplementary material. Further inquiries can be directed to the corresponding author.
